# Modeling healthcare resource dynamics and its application based on interregional population mobility

**DOI:** 10.3389/fpubh.2025.1582024

**Published:** 2025-07-02

**Authors:** Jiaming Guo, Beibei Yan, Yanli Yang, Yifei Ning, Yan Zhou, Rui Zhang, Yifei Ma, Jiantao Li, Hongmei Yu, Jun Xie

**Affiliations:** ^1^School of Public Health, Shanxi Medical University, Taiyuan, China; ^2^Third Hospital of Shanxi Medical University, Shanxi Medical University, Shanxi Bethune Hospital, Shanxi Academy of Medical Sciences, Tongji Shanxi Hospital, Taiyuan, China; ^3^School of Management, Shanxi Medical University, Taiyuan, China; ^4^Shanxi Provincial Key Laboratory of Major Diseases Risk Assessment, Shanxi Medical University, Taiyuan, China; ^5^Center of Reverse Microbial Etiology, Shanxi Medical University, Taiyuan, China; ^6^MOE Key Laboratory of Coal Environmental Pathogenicity and Prevention, Shanxi Medical University, Taiyuan, China; ^7^Department of Biochemistry and Molecular Biology, Shanxi Medical University, Taiyuan, China; ^8^Shanxi Key Laboratory of Birth Defect and Cell Regeneration, Shanxi Medical University, Taiyuan, China

**Keywords:** infectious disease prevention and control, population mobility, healthcare resource congestion, healthcare resource dynamical model, SARS-COV-2

## Abstract

**Objective:**

This study aims to assess the impact of inter-regional population mobility on epidemic progression and healthcare resource congestion during acute infectious disease outbreaks, providing a scientific basis for population control and healthcare resource allocation during pandemics.

**Methods:**

Using the SARS-CoV-2 pandemic as a case study, we selected two city pairs—“Taiyuan–Jinzhong” and “Linfen–Yuncheng” as research subjects. Based on real SARS-CoV-2 transmission data and the Baidu Migration Index, we constructed a dynamic healthcare resource model incorporating population mobility factors. We quantified epidemic transmission and healthcare resource congestion using three indicators: cumulative cases, the onset time of healthcare resource congestion, and its duration. By analyzing these metrics, we explored the effect of migration rates on infection scale and healthcare resource congestion.

**Results:**

The model-fitted curves closely aligned with the actual data, with most observed data points falling within the 95% confidence interval. Our results suggest that population mobility affects both cumulative cases and healthcare resource congestion, with variation between regions. Unidirectional restrictions on population movement reduced cumulative cases in the outflow region, delayed the onset of healthcare resource congestion and shortened its duration; however, they also increased cumulative cases in the inflow region, advanced the onset of healthcare resource congestion and prolonged its duration. Bidirectional movement restrictions increased cumulative cases in high-prevalence regions, but did not change the onset time of healthcare resource congestion and either maintained or increased its duration. In contrast, in low-prevalence regions, bidirectional restrictions reduced cumulative cases, maintained the onset time of healthcare resource congestion, and either shortened or maintained its duration.

**Conclusion:**

The healthcare resource dynamics model provides an effective framework for simulating the interplay between population mobility, epidemic transmission, and the congestion on medical resources. In the event of an infectious disease outbreak, this model can be integrated into a regionally unified platform for healthcare resource allocation. By incorporating real-time epidemic data and the healthcare capacities of different areas, the model enables targeted interventions based on the principles of zoning, classification, and time-based management. This approach helps to contain the spread of the epidemic, ease pressure on medical systems, and minimize socio-economic disruptions.

## 1 Introduction

The outbreak and spread of infectious diseases can lead to a rapid surge in patient numbers in a short period, resulting in shortages and congestion on healthcare resources (1–4). Once the healthcare system becomes congested, the increasing number of new cases may not receive timely treatment or effective isolation, which not only hinders the effective treatment of the disease but also exacerbates the congestion on healthcare resources and accelerates epidemic transmission. When healthcare resources are congested, mortality rates tend to rise. Brizzi et al. (5) assessed the geographic and temporal fluctuations in hospitalization mortality due to SARS-CoV-2 in Brazil using a Bayesian mortality model, revealing that resource shortages were a significant factor contributing to high mortality. Additionally, studies by Ji et al. (6) found a correlation between SARS-CoV-2 mortality rates and healthcare resource availability. Therefore, effective allocation of healthcare resources during an epidemic to accommodate the surge in patient numbers is crucial for avoiding or alleviating healthcare resource congestion. Population mobility affects the speed of disease transmission between regions, thereby expanding the scale of the epidemic (7, 8). Zhao et al. (7) used a logistic function to show that an increase in new cases in the outflow region is positively correlated with an increase in new cases in the inflow region, highlighting the importance of travel restrictions in slowing virus spread. Kraemer et al. (8) constructed a generalized linear model for SARS-CoV-2, showing that travel restrictions can control the transmission rate of the virus.

Dynamical models of infectious diseases are typically based on the different states of individuals or populations (such as susceptible, infected, recovered, etc.) and the transition probabilities and rates between these states to simulate the dynamics of disease transmission at different points in time. These models have been widely used to predict disease spread (9, 10). For example, Yang et al. (9) developed a dynamical model based on the actual situation in Xinjiang, China, to predict the evolution of the epidemic and evaluate control measures. Aronna et al. (10) modified the traditional susceptible-infected-recovered (SIR) model to study the effectiveness of non-pharmaceutical interventions in controlling the epidemic. Although these methods effectively simulate transitions between different states within the population, they overlook the important influence of geographic distribution and population mobility on disease transmission. Other studies have used patch model to investigate the impact of population mobility on disease spread between different regions (11–13). For example, Hsieh et al. (11) constructed a two-patch model to study the spread of influenza between two regions and showed that controlling the migration of new cases from low-prevalence to high-prevalence areas could delay virus transmission. Das et al. (12) built the patch model for three regions in India and analyzed the impact of population mobility on these regions. The study found that isolation of high-prevalence areas led to continued new cases in those areas while reducing infections in other areas, suggesting that travel restrictions are critical to controlling SARS-CoV-2 transmission. Cui et al. (13) used the patch-model to study the impact of population movement between different regions on disease transmission, and they found that population mobility expands the infection range between two regions, leading to the increase in both the number and proportion of patients. Compared with traditional dynamical models, the patch model capture spatial dynamics and population movement patterns, allowing for a quantitative analysis of how population mobility affects epidemic trends. Although infectious disease dynamics models have been widely used to predict the progression of epidemics across different regions, there remains a lack of systematic analysis concerning the allocation of healthcare resources at the regional level. During outbreaks, population mobility can accelerate the spread of disease, resulting in the overburdening and congestion of healthcare systems (14). Therefore, it is essential to develop an analytical framework that incorporates the dynamics of inter-regional population movement to support the formulation of more scientifically grounded prevention and control strategies. Such a model can facilitate the rational allocation of healthcare resources across regions and is of great significance in enhancing both medical treatment capacity and the overall efficiency of public health responses during epidemic emergencies.

This study aims to assess the impact of population mobility on epidemic transmission trends and healthcare resource congestion, evaluate the ability of healthcare systems in different regions to respond to epidemic shocks, and optimize healthcare resource allocation based on population mobility characteristics. First, based on the Baidu Migration Index and real SARS-CoV-2 data, the study identifies Taiyuan and Jinzhong in central Shanxi Province as the regions with the largest bidirectional population mobility, and Linfen and Yuncheng in southeastern Shanxi Province as the regions with the largest bidirectional population mobility. Considering the population mobility characteristics of these regions and the mechanisms of infectious disease transmission, we constructed a population mobility-based healthcare resource dynamical model to simulate the epidemic spread dynamics and changes in healthcare resource congestion under actual population mobility scales within Shanxi Province. Finally, we performed the sensitivity analysis to compare the changes in healthcare resource congestion in two groups of cities under different population mobility patterns (i.e., unidirectional and bidirectional flow) and optimized specific healthcare resource allocation plans. The main assumptions of the study are as follows:

1. Transmissions from asymptomatically infected individuals was excluded.
2. Home isolation is assumed to be non-infectious, as it is unlikely to trigger community transmission during the isolation period.
3. Individuals who have already been recovered will not become reinfected in the short term.
4. The capacity of healthcare resources (e.g., number of resources) and contact rates varied as the pandemic evolved. During the initial period after the liberalization of outbreak control in a region, infection rates gradually peaked, and the rapid increase in demand for hospital care corresponded to the increased demand for healthcare resources, marking the moment in which the outbreak peaked. During this period, social interactions and person-to-person contact rates declined. Therefore, parameters for healthcare resource capacity, contact rates, and hospitalization rates were changed at the same time.
5. As most individuals did not undergo SARS-CoV-2 PCR testing after control measures were released, we considered those with SARS-CoV-2-like symptoms (e.g., a fever or cough) as new cases.
6. Death only occurred among hospitalization.
7. If sufficient healthcare resources are available, there will also be enough medical staff and equipment.
8. Available hospital beds of zero indicates that congestion has begun to occur.

## 2 Method

Cumulative cases and healthcare resource congestion (including onset time and congestion duration) are used as quantitative indicators to assess the level of epidemic control. Utilizing the Baidu Migration Index and real SARS-CoV-2 infection data from Shanxi Province, this study examines the impact of population mobility on cumulative infection numbers and healthcare resource congestion of two city pairs: Taiyuan–Jinzhong and Linfen–Yuncheng. Building on the traditional SEIR patch model, we introduce three additional compartments: quarantined individuals (Q), hospitalizations (H), and deceased individuals (D). In addition, population migration rates and contact rates are incorporated to characterize population mobility, forming a healthcare resource dynamical model based on population mobility. The model parameters are estimated and fitted using Markov Chain Monte Carlo (MCMC) (14–16) to simulate the transmission dynamics of SARS-CoV-2. Through sensitivity analysis, this study investigates the impact of population mobility on regional epidemic control and healthcare resource congestion. By comparing healthcare resource conditions between the two city pairs during the epidemic, the study reveals how public health interventions and their intensity (i.e. unidirectional vs. bidirectional population movement control) affect healthcare resource congestion under different city sizes and healthcare resource capacities. The findings provide theoretical support for improving public health emergency management systems in China and globally.

### 2.1 Data sources

The daily number of new cases was estimated based on the infection rate for each city provided by the Shanxi Provincial Center for Disease Control and Prevention (CDC) and calculated using the “total local population incidence rate.” Data on hospitalizations were obtained from the designated hospital monitoring system of the Shanxi Provincial Health Commission. The total number of hospital healthcare resources in each city in Shanxi Province was obtained from the “14th Five-Year Plan for Medical and Health Service System Development of Shanxi Province.” Population migration data were processed based on the Baidu Migration Scale Index (17, 18). According to Wang and Yan (17), there is a functional relationship between the population migration index published by “Baidu Huiyan” and the actual population mobility. Specifically, at a given date *t*, the migration scale index from any region *i* to region *j* is linearly correlated with the actual number of migrating individuals, with a scale factor of 3.24 × 10^−5^, with the specific mathematical expression *M*_*ij*_ = 3.24 × 10^−5^^*^H_*ij*_ (*M*_*ij*_ refers to the Baidu index of migration from region *i* to region *j* at time *t*, and *H*_*ij*_ refers to the actual number of people migrating from region *i* to region *j* at time *t*). Based on the Baidu index from December 9, 2022 to January 13, 2023 for the municipal administrative districts in Shanxi province index data (18) and applying the above functional relationship, we estimated the average daily population mobility between Taiyuan, Jinzhong, Linfen and Yuncheng. Subsequently, we calculated the corresponding migration rates. Explanations of the migration rate and specific values are presented in Tables 1, 2, respectively. The resident population of the four selected cities in Shanxi Province at the end of 2022 was obtained from the official website of the Shanxi Bureau of Statistics (19).

**Table 1:** Model symbol interpretation table.

**Parameters**		**Define**
*c*_*i*_(*t*)	*c*_0*i*_	Initial contact rate in area *i*
	*c*_*bj*_	Minimum contact rate after the outbreak of the epidemic in area *i*
δ_*i*_		The potential decline rate of contact rate in area *i*
β_*i*_		Probability of an infected individual transmitting the infection through contact in area *i*
$\frac{1}{\omega_{i}}$		Average duration of latent period in area *i*
*m*_*i*_		The conversion rate from infected individuals to home-isolated infected individuals in area *i*
θ_*i*_		The conversion rate from home-isolated infected individuals to hospitalized individuals in area *i*
*H*_*ci*_ (*t*)	*H*_0*i*_	Initial number of respiratory beds in area *i*
	*H*_*mi*_	Maximum number of respiratory beds in area *i*
*T*_1_		Onset time of increased healthcare resources in area *i*
δ_1*i*_		The allocation rate of healthcare resources in area *i*
α_1*i*_		The case fatality rate of infected individuals in area *i*
α_2*i*_		The case fatality rate of home-isolated infected individuals in area *i*
α_3*i*_		The case fatality rate of hospitalized individuals in area *i*
1/γ_1*i*_		Recovery time for infected individuals in area *i*
1/γ_2*i*_		Recovery time for home-isolated infected individuals in area *i*
1/γ_3*i*_		Recovery time for hospitalized individuals in area *i*
*a*_ij_		Migration rate from region *j* to region *i*
*Initial value*		Description
*S*_*i*_(0)		Initial susceptible individuals
*E_*i*_* (0)		Initial exposed individuals
*I_*i*_* (0)		Initial infected individuals
*Q_*i*_* (0)		Initial home-isolated infected individuals
*H_*i*_* (0)		Initial hospitalized individuals
*R_*i*_* (0)		Initial recovered individuals
*D_*i*_* (0)		Initial dead individuals
*N_*i*_*		Total number of individuals in area *i*

**Table 2:** Taiyuan (*i* = 1) Jinzhong (*j* = 2) Linfen (*i* = 3) Yuncheng (*j* = 4) parameter list.

**Parameter**	**First stage value taking**	**Second stage value taking**	**Source**
β_1_	0.01	0.01	Real epidemic situation/MCMC
β_2_	0.02	0.02	Real epidemic situation/MCMC
β_3_	0.01	0.01	Real epidemic situation/MCMC
β_4_	0.01	0.01	Real epidemic situation/MCMC
γ_11_	0.33	0.33	Real epidemic situation
γ_21_	0.11	0.11	Real epidemic situation
γ_31_	0.1	0.1	Real epidemic situation
γ_12_	0.2	0.2	Real epidemic situation
γ_22_	0.1	0.1	Real epidemic situation
γ_32_	0.1	0.1	Real epidemic situation
θ_1_	0.03	1	Real epidemic situation
θ_2_	0.01	0.24	Real epidemic situation
*a*_12_	0.01	0.01	Baidu Migration Index
*a*_21_	0.01	0.01	Baidu Migration Index
α_31_	0.01	0	Real epidemic situation
α_32_	0.01	0.01	Real epidemic situation
*c*_01_	50	50	MCMC
*c*_02_	43	43	MCMC
*c*_*b*1_	/	24	Real epidemic situation
*c*_*b*2_	/	10	Real epidemic situation
δ_11_	/	0.5	Real epidemic situation
δ_12_	/	0.01	Real epidemic situation
δ_2_	/	0.27	Real epidemic situation
δ_1_	/	0.42	real epidemic situation
*H*_01_	1,697	1,697	[4]
*H*_02_	1,330	1,330	[4]
*H*_*m*1_	33,969	33,969	[4]
*H*_*m*2_	21,216	21,216	[4]
*m*_1_	0.08	0.30	Real epidemic situation
*m*_2_	0.90	1	Real epidemic situation
*N*_1_	5,435,000	5,435,000	Official data
*N*_2_	3,394,500	3,394,500	Official data
ω	1.52	1.52	Real epidemic situation
γ_13_	0.26	0.26	Real epidemic situation
γ_23_	0.20	0.20	Real epidemic situation
γ_33_	0.10	0.10	Real epidemic situation
γ_14_	0.20	0.20	Real epidemic situation
γ_24_	0.10	0.20	Real epidemic situation
γ_34_	0.07	0.10	Real epidemic situation
θ_3_	0.43	0.80	Real epidemic situation
θ_4_	0.03	0.52	Real epidemic situation
*a*_34_	0.005	0.006	Baidu Migration Index
*a*_43_	0.004	0.005	Baidu Migration Index
α_33_	0.001	0	Real epidemic situation
α_34_	0.001	0.007	Real epidemic situation
*c*_03_	64	64	MCMC
*c*_04_	55	55	MCMC
*c*_*b*3_	23	23	Real epidemic situation
*c*_*b*4_	15	15	Real epidemic situation
*H*_03_	839	839	[4]
*H*_*m*3_	24,416	24,416	[4]
*m*_3_	0.02	0.52	Real epidemic situation
*m*_4_	0.5	0.68	Real epidemic situation
*N*_3_	3,906,600	3,906,600	Actual circumstances
*T*	11	11	Actual circumstances
*H*_*m*4_	29,491	29,491	[4]
*N*_4_	4,718,500	4,718,500	Official data
δ_3_	/	0.36	Real epidemic situation
δ_4_	/	0.39	Real epidemic situation
δ_13_	/	0.002	Real epidemic situation
δ_14_	/	0.003	Real epidemic situation
*H*_04_	911	911	[4]

### 2.2 Construction of the healthcare resource dynamical model based on population mobility

**Figure 1:**
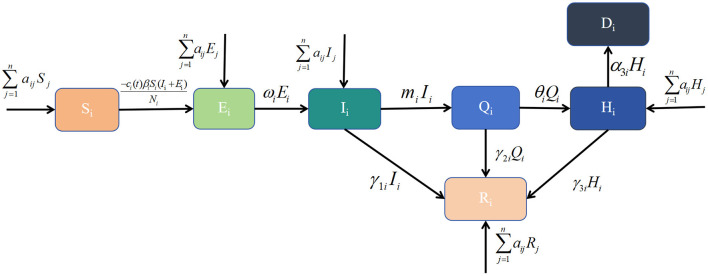
Transmission diagram of Omicron.

Common dynamical models include the SI, SIR and SEIR models. The patch model, widely used in ecology, examines interactions between biological populations and their environment, as well as interactions among different populations (20). Considering real conditions and available data, this study integrates the patch model with dynamical models to develop a healthcare resource dynamics model based on population mobility. This model is used to analyse the epidemic scale and the dynamic changes in healthcare resources. Based on the pathological characteristics of infectious diseases and the real epidemic situation, the population in each region is divided into seven compartments: susceptible individuals (*S*_*i*_), exposed individuals (*E*_*i*_), symptomatic infected individuals (*I*_*i*_), home-isolated infected individuals (*Q*_*i*_), recovered individuals (*R*_*i*_), hospitalized individuals (*H*_*i*_), and deceased individuals (*D*_*i*_). Their definitions are as follows, *S*_*i*_ denotes those who have not been infected and lack immunity to the virus, making them vulnerable to infection. *E*_*i*_ denotes those who have been infected but have not yet developed symptoms. *I*_*i*_ denotes those who have been infected with the virus and exhibit symptoms, making them infectious. *Q*_*i*_ denotes those who have been infected and developed symptoms but have mild illness and choose to isolate at home. *H*_*i*_ denotes those with severe symptoms or requiring specialized treatment who seek medical care at hospitals. *R*_*i*_ denotes those who have recovered from the infection and developed temporary immunity, preventing reinfection for a certain period. *D*_*i*_ denotes those who have died due to the infection. Additional parameter definitions are provided in Table 1. The transitions between these compartments can be described as follows: *S*_*i*_ may become *E*_*i*_, *E*_*i*_ may progress to *I*_*i*_ after the latent period; *I*_*i*_may recover (*R*_*i*_), deteriorate into severe cases requiring hospitalization (*H*_*i*_), or die (*D*_*i*_) due to lack of timely medical intervention; home-isolated individuals (*Q*_*i*_) may either recover (*R*_*i*_), progress to hospitalization (*H*_*i*_) if their condition worsens, or die (*D*_*i*_); hospitalized patients (*H*_*i*_) may either recover (*R*_*i*_) and be discharged or succumb to the disease (*D*_*i*_). At time *t*, healthcare resources available in region i are represented by *H*_*ci*_(*t*). Population movement occurs between regions *i* and *j*, affecting the numbers of susceptible, exposed, infected, hospitalized, and recovered individuals. These transitions, along with other model assumptions, are illustrated in Figure 1 and lead to the derivation of the following dynamic healthcare resource model based on population mobility:


(1) $$\left\{ \begin{array}{l} \;\frac{dS_{i}}{dt}=\frac{-c_{i}(t)\beta_{i}S_{i}(I_{i}+E_{i})}{N_{i}}+\sum_{j=1}^{n}a_{ij}S_{j}\\ \;\frac{dE_{i}}{dt}=\frac{c_{i}(t)\beta_{i}S_{i}(I_{i}+E_{i})}{N_{i}}-\omega_{i}E_{i}+\sum_{j=1}^{n}a_{ij}E_{j}\\ \;\frac{dI_{i}}{dt}=\omega_{i}E_{i}-m_{i}I_{i}-\gamma_{1i}I_{i}+\sum_{j=1}^{n}a_{ij}I_{j}\\ \;\frac{dQ_{i}}{dt}=m_{i}I_{i}-\min\{\theta_{i}Q_{i},\max\{(H_{ci}(t)-H_{i}),0\}\}-\gamma_{2}Q_{i}\\ \frac{dH_{i}}{dt}=\min\{\theta_{i}Q,\max\{(H_{ci}(t)-H_{i}),0\}\}-\alpha_{3i}H_{i}-\gamma_{3i}H_{i}+\sum_{j=1}^{n}a_{ij}H_{j}\\ \;\frac{dD_{i}}{dt}=\alpha_{3i}H_{i}\\ \;\frac{dR_{i}}{dt}=\gamma_{1i}I_{i}+\gamma_{2i}Q_{i}+\gamma_{3i}H_{i}+\sum_{j=1}^{n}a_{ij}R_{j} \end{array}\right.$$

with $i=\;1^{∼}n$, $\overset{n}{\underset{i=1}{\sum }}a_{ij}=0$. When new policies related to the SARS-CoV-2 pandemic came into effect in December 2023, restrictions on population mobility were lifted, leading to an increase in contact rates among individuals and a rapid short-term spread of the pandemic. Subsequently, most individuals began self-isolate at home or receiving treatment in hospitals, gradually reducing the contact rate. Therefore, the contact rate is a function that decreases over time *t* (7), as shown below,


(2) $$\begin{array}{l} c_{i}(t)=\{\begin{array}{l} c_{0i}\;t\leq T_{1},\\ (c_{0i}-c_{bi})e^{-\delta_{1i}(t-T_{1})}+c_{bi}\;>T_{1}. \end{array} \end{array}$$

where *c*_0*i*_ denotes the contact rate at the initial time and *c*_0*i*_ = *c*_*i*_(0). *c*_*bi*_ denotes the minimum contact rate given the current control strategy $\underset{t\to \infty }{\lim}c\left(t\right)=c_{b}$. denotes that the contact rate decreases exponentially.

The increase and decrease in the number of available hospital beds are used as quantitative indicators to reflect the dynamic allocation capacity of healthcare resources (21). The onset, duration, and end of healthcare resource congestion are quantified in terms of hospital bed congestion, including their onset time, duration, and end time. As the government gradually expands healthcare resources, particularly by increasing supply in response to the pandemic, we use a Logistic growth model to simulate changes in the availability of healthcare resources (21), as follows:


(3) $$\frac{H_{ci}(t)}{dt}=\delta_{i}H_{ci}(t)[1-\frac{H_{ci}(t)}{H_{mi}}].$$

where δ_*i*_ represents the capacity for hospital bed production and allocation, while *H*_*mi*_ denotes the maximum number of available beds during the pandemic. These two factors indicate the region's capacity to respond to an outbreak. In the early stages of the pandemic, hospital healthcare resources often remain relatively stable. This may be due to lower hospitalization demand or a lack of clear understanding of the required healthcare resources. Therefore, by solving the previously mentioned logistic equation, we can use the following piece-wise function to calculate the daily availability of healthcare resources (21),


(4) $$H_{ci}(t)=\left\{ \begin{array}{l} H_{0i}\;t\leq T_{1},\\ frac(H_{0i}H_{mi})H_{oi}+(H_{mi}-H_{oi})e^{-\delta_{i}(t-T_{i})}t>T_{1}.\; \end{array}\right.$$

where *H*_0*i*_ represents the initial number of beds at the time of the outbreak, and *T*_*i*_ represents the critical point at which the city began to add healthcare resources, including beds. New cases may not be able to be hospitalized due to beds' congestion, therefore, min{θ_*i*_*Q*_*i*_, max{*H*_*ci*_(*t*)−*H*_*i*_, 0}} is used to describe the number of new hospitalizations per day, which is a partition function based on the relationship between θ_1_*Q* and where θ_1_*Q* is the number of confirmed cases requiring hospital beds on the day *t*. *H*_*ci*_(*t*) is the number of hospital beds on day *t*. Therefore, *H*_*i*_(*t*) = max {*H*_*ci*_(*t*)−θ_*i*_*Q*_*i*_, 0} is the number of available beds in that day.

### 2.3 Parameter estimation and data fitting of model

In this part, we first fitted the daily cumulative number of cases and hospitalizations reported in Taiyuan–Jinzhong and Linfen–Yuncheng from 9 December 2022 to 13 January 2023 in stages using **Equation 1**. Latin hypercube sampling and Markov chain Monte Carlo (MCMC) simulations (14–16) were used to estimate the unknown parameters β_1_, β_2_, β_3_, β_4_, *c*_01_, *c*_02_, *c*_03_, *and c*_04_. The ODE45 function in MATLAB software was used to calculate the number of daily beds in the target city using **Equation 4** to analyze the changes in the onset and duration of the healthcare resource congestion. Finally, we perform sensitivity analysis using Equations 1–4.

## 3 Result

### 3.1 Parameter estimation

First, we estimate the initial values of several key parameters. Their descriptions, numerical values, and data sources are summarized in Table 3. The detailed procedures for calculating these parameters are provided in Section 3.1 (1)–(5).

[1] From the questionnaire survey, there were 8,033 new cases and 77 hospitalizations in Taiyuan on 9 December 2022, and the latent period was 1.52 days (3, 16) (*H*_1_(0) = 77, ω = 1.52), so the number of patients with latent period at the initial time *E*_1_(0) = 22, 472. At the same time, it is assumed that the number of home quarantines, the number of deaths, and the number of recoveries at the initial time are all 0, i.e., *Q*_1_ = 0, *D*_1_ = 0, *R*_1_ = 0. This gives the initial value of 5,404,418 susceptible individuals (*S*_1_(0) = 5, 435, 000−*E*_1_(0)−*I*_1_−*H*_1_(0) = 5, 404, 418).
[2] Similarly, on 9 December 2022, there are 2,867 new cases, 69 hospitalizations, and 9,109 latent patients at the initial time in Jinzhong (*I*_2_(0) = 2, 867, *H*_2_(0) = 69, *E*_2_(0) = 9, 109). It is also assumed that the number of home quarantine, deaths, and recoveries at the initial time is 0, i.e., *Q*_2_ = 0, *D*_2_ = 0, *R*_2_ = 0. This gives the initial value of 3,382,455 susceptible individuals (*S*_2_(0) = 3, 394, 500−*E*_2_(0)−*I*_2_−*H*_2_(0) = 3, 382, 455).
[3] Similarly, there are 3,274 new cases, 61 hospitalizations, and 9,728 latent patients at the initial moment in Linfen on 9 December 2022 (*I*_3_(0) = 3, 274, *H*_3_(0) = 61, *E*_3_(0) = 9, 728). It is also assumed that the number of home quarantine, deaths, and recoveries at the initial time is 0, i.e., *Q*_3_ = 0, *D*_3_ = 0, *R*_3_ = 0. This gives the initial value of 3,893,537 susceptible individuals (*S*_3_(0) = 3, 906, 600−*E*_3_(0)−*I*_3_−*H*_3_(0) = 3, 893, 537).
[4] Similarly, at the initial moment in Yuncheng on 9 December 2022 there were 5,558 new cases, 78 hospitalizations, and 17,659 latent patients (, *H*_4_(0) = 78, *E*_4_(0) = 17, 659). It is also assumed that the number of home quarantine, deaths, and recoveries at the initial moment is 0, i.e., *Q*_4_ = 0, *D*_4_ = 0, *R*_4_ = 0. This gives the initial value of 4,695,205 susceptible individuals (*S*_4_(0) = 4, 718, 500−*E*_4_(0)−*I*_4_−*H*_4_(0) = 4, 695, 205). According to the “14th Five-Year Plan of Medical and Health Service System of Shanxi Province” (2024 Revision), the number of beds in medical and healthcare institutions per 1,000 resident population in Shanxi Province is ~6.25, and the bed-to-person (medical and technical staff) ratio is 1:1.62. It can be estimated that the number of beds in hospitals in Taiyuan City is 33,969 (*H*_*m*1_ = 6.25 × 543, 500), with ~1,697 beds at respiratory department (*H*_01_ = 33, 969 × 123÷2, 462) (22); the number of beds in Jinzhong City Hospital was 21,216 (*H*_*m*2_ = 6.25 × 3, 394, 500), and the number of beds at respiratory department was about 408 (*H*_02_ = 21, 216 × 94÷1, 500) (23); the total number of beds in Linfen City Hospital was 24,416 (*H*_*m*3_ = 6.25 × 3, 906, 600), and the number of medical beds at respiratory department was 839 (*H*_03_ = 24, 416 × 68÷1, 980) (24); the number of hospital beds in Yuncheng City Hospital was 29,491 (*H*_*m*4_ = 6.25 × 4, 718, 500), and the number of total medical beds at respiratory department was 910 (*H*_04_ = 29, 491 × 61÷1, 975) (25).

**Table 3:** Initial parameter values table.

**Parameter**	**Description**	**Value**	**Source**
*I_1_*(0)	Initial infected individuals in Taiyuan	8,033	[1]
*H_1_*(0)	Initial hospitalized individuals in Taiyuan	77	[1]
*E_1_*(0)	Initial exposed individuals in Taiyuan	22,472	[1]
*Q_1_*(0)	Initial home-isolated infected individuals in Taiyuan	0	[1]
*D_1_*(0)	Initial dead individuals in Taiyuan	0	[1]
*R_1_*(0)	Initial recovered individuals in Taiyuan	0	[1]
*S_1_*(0)	Initial susceptible individuals in Taiyuan	5,404,418	[1]
*I_2_*(0)	Initial infected individuals in Jinzhong	2,867	[2]
*H_2_*(0)	Initial hospitalized individuals in Jinzhong	69	[2]
*E_2_*(0)	Initial exposed individuals in Jinzhong	9,109	[2]
*Q_2_*(0)	Initial home-isolated infected individuals in Jinzhong	0	[2]
*D_2_*(0)	Initial dead individuals in Jinzhong	0	[2]
*R_2_*(0)	Initial recovered individuals in Jinzhong	0	[2]
*S_2_*(0)	Initial susceptible individuals in Jinzhong	3,382,455	[2]
*I_3_*(0)	Initial infected individuals in Linfen	3,274	[3]
*H_3_*(0)	Initial hospitalized individuals in Linfen	77	[3]
*E_3_*(0)	Initial exposed individuals in Linfen	9,278	[3]
*Q_3_*(0)	Initial home-isolated infected individuals in Linfen	0	[3]
*D_3_*(0)	Initial dead individuals in Linfen	0	[3]
*R_3_*(0)	Initial recovered individuals in Linfen	0	[3]
*S_3_*(0)	Initial susceptible individuals in Linfen	3,893,537	[3]
*I_4_*(0)	Initial infected individuals in Yuncheng	5,558	[4]
*H_4_*(0)	Initial hospitalized individuals in Yuncheng	78	[4]
*E_4_*(0)	Initial exposed individuals in Yuncheng	17,659	[4]
*Q_4_*(0)	Initial home-isolated infected individuals in Yuncheng	0	[4]
*D_4_*(0)	Initial dead individuals in Yuncheng	0	[4]
*R_4_*(0)	Initial recovered individuals in Yuncheng	0	[4]
*S_4_*(0)	Initial susceptible individuals in Yuncheng	4,695,205	[4]

### 3.2 Simulation and calculation of cumulative cases and hospitalizations

**Figure 2:**
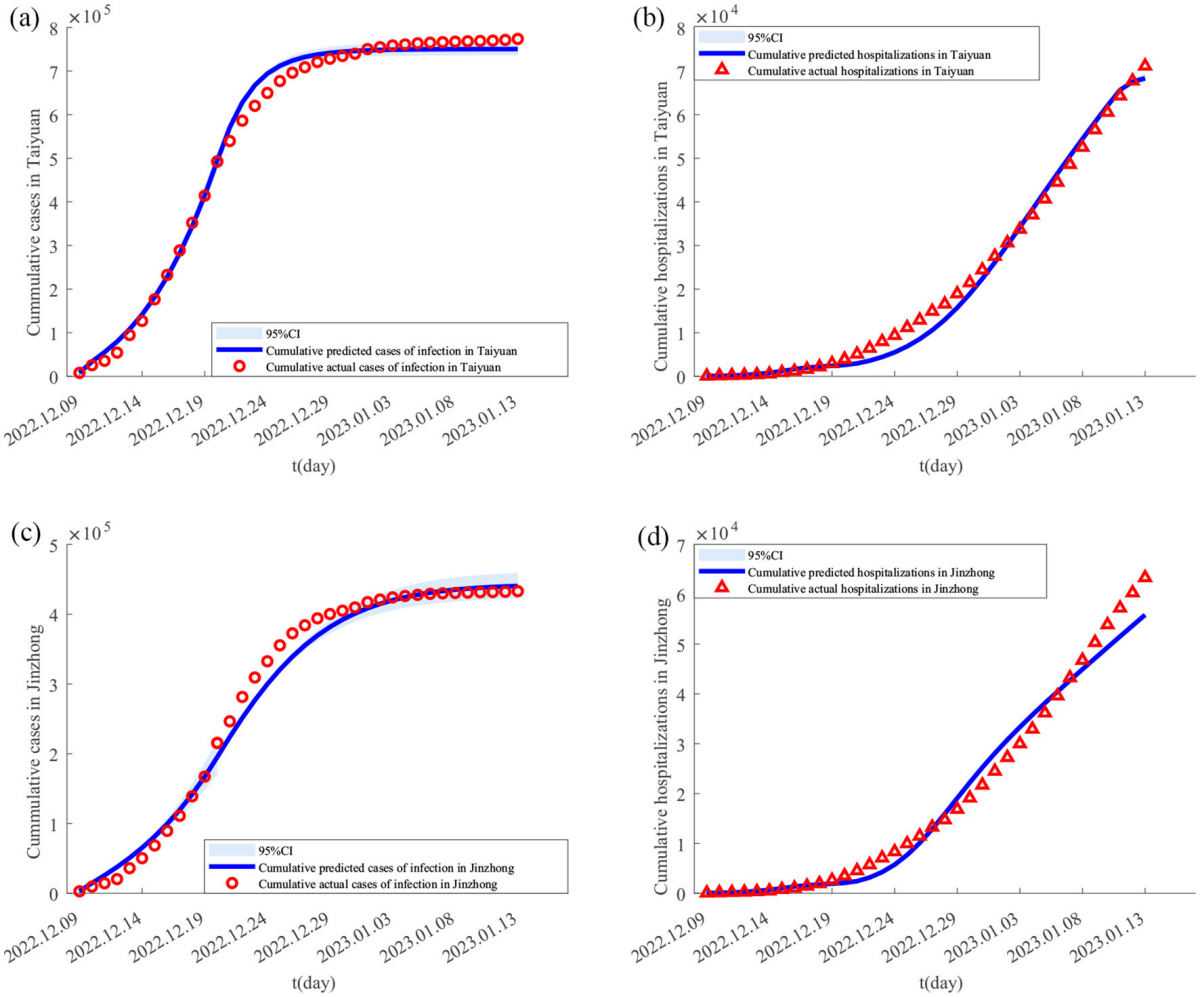
Fitting results of cumulative new cases and hospitalizations in Taiyuan and Jinzhong from December 9, 2022 to January 13, 2023. In **(A–D)** the solid lines represent the fitted cumulative cases and hospitalizations in Taiyuan and Jinzhong, respectively; the circles indicate the corresponding reported data; and the shaded areas show the 95% confidence intervals.

**Figure 3:**
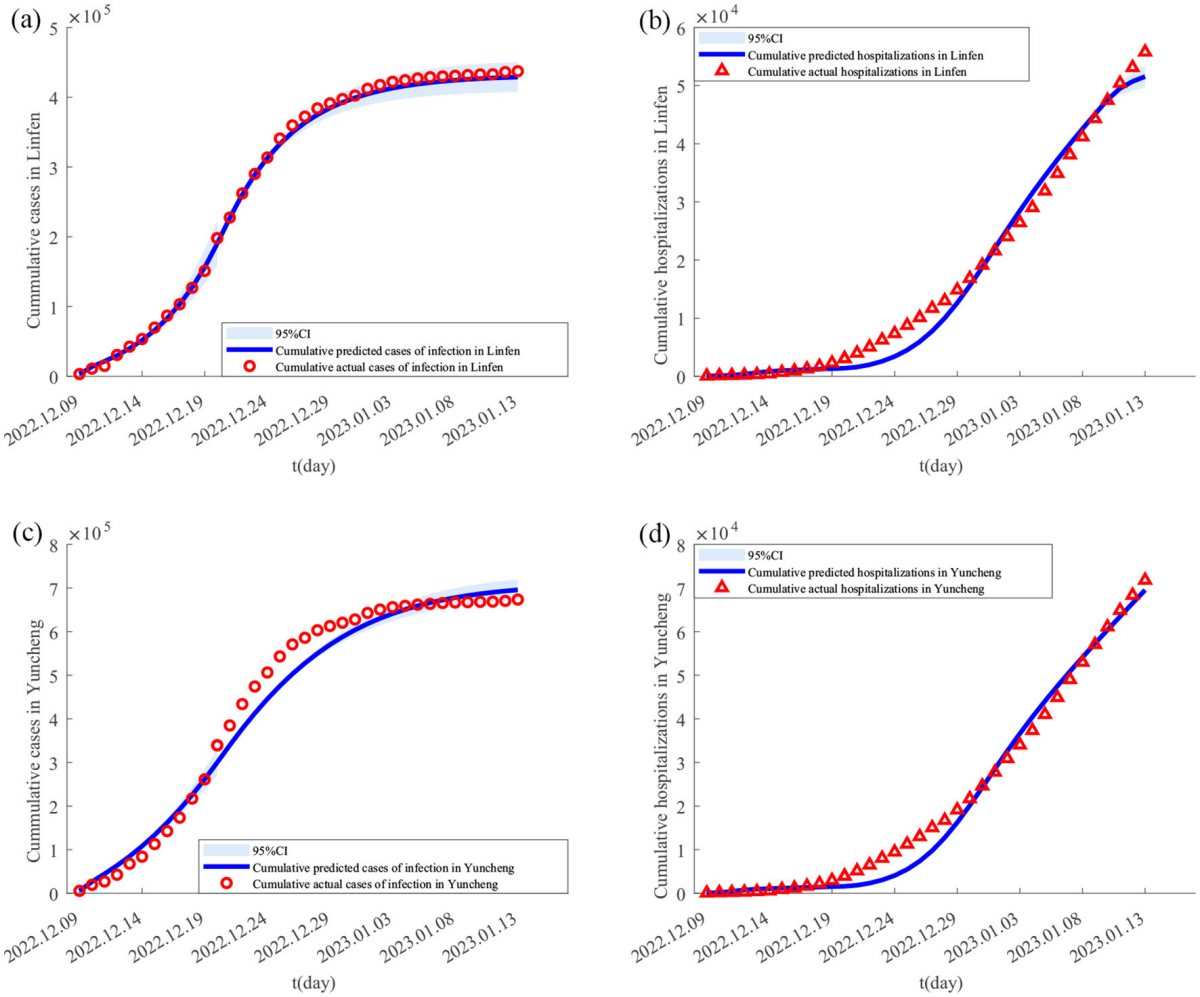
Fitting results of cumulative new cases and hospitalizations in Yuncheng and Linfen from December 9, 2022 to January 13, 2023. In **(A–D)** the solid lines represent the fitted cumulative cases and hospitalizations in Yuncheng and Linfen, respectively; the circles indicate the corresponding reported data; and the shaded areas show the 95% confidence intervals.

**Figure 4:**
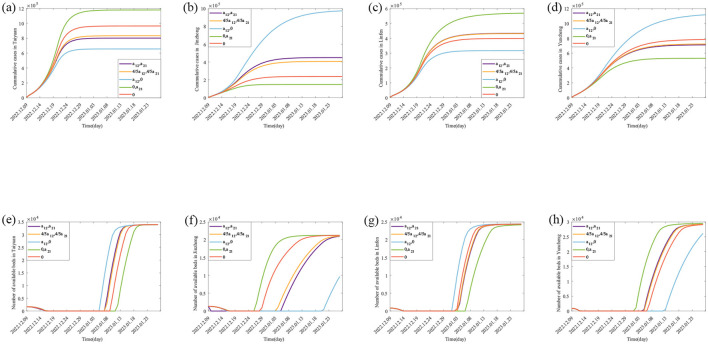
Theoretically, the number of Final cumulative numbers of new cases and daily potential empty beds under different scenarios. **(A–D)** The changes in the cumulative cases in Taiyuan, Jinzhong, Linfen, and Yuncheng under various scenarios; **(E–H)** represents the daily potential empty beds in Taiyuan, Jinzhong, Linfen, and Yuncheng under various scenarios.

Using the Monte Carlo Markov Chain (MCMC) algorithm, **Equation 1** was simulated 4,000 times to obtain the parameter estimates for the Taiyuan–Jinzhong and Linfen–Yuncheng pairs (see Table 3). The solutions to **Equation 1** for these two pairs, along with their confidence intervals, are shown in Figures 2, 3. From Figure 2, it can be observed that the fitted curves for daily cumulative cases and cumulative hospitalizations in Taiyuan and Jinzhong closely match the actual trajectory. Similarly, Figure 3 shows that the fitted curves for daily cumulative cases and cumulative hospitalizations in Linfen and Yuncheng align well with the real data. All parameter values related to the calculations are listed in Table 3. To compute the daily changes in cumulative infections under real conditions, we used the ODE45 function in MATLAB along with **Equation 1**. The results are depicted as solid purple lines in Figures 4a–d. Based on the calculations, the final cumulative infection counts for Taiyuan, Jinzhong, Linfen, and Yuncheng were 801,891, 449,387, 433,293, and 710,766, respectively.

### 3.3 Simulation and calculation of daily available hospital beds

The parameter values in Table 3 are substituted into Equation 4, and MATLAB software is used to generate the purple lines in Figures 4e, f, which are the real daily spare healthcare resource dynamics of the two pairs of cities. According to the purple solid lines in Figures 4e, f, the healthcare resource congestion in Taiyuan City and the healthcare resource congestion in Jinzhong City both started on 17 December, and the healthcare resource congestion in Taiyuan City lasted for 22 days and ended on 7 January; whereas the healthcare resource congestion in Jinzhong City lasted for 20 days and ended on 5 January, and the healthcare resource congestion in Jinzhong City was alleviated 2 days earlier than that in Taiyuan City. According to the purple solid line in Figure 4f, the healthcare resource congestion in Linfen City started on 14 December and lasted for 21 days, ending on 3 January; according to the purple solid line in Figure 4g, the healthcare resource congestion in Yuncheng City started on 13 December and lasted for 23 days, ending on 4 January; compared with Yuncheng City, the healthcare resource congestion in Linfen City ended 1 day earlier than that in Yuncheng City.

### 3.4 Sensitivity analysis

4In this section, we analyse the effect of population migration rates on the onset and end of healthcare resource congestion and their duration. First, we construct two different scenarios: unidirectional population migration control (i.e. only population migration from *i* to *j* or only population migration from *j* to *i*) and bidirectional population migration control (i.e. the rate of population migration between *i* and *j* is reduced to 4/5 of the actual situation and population migration between *i* and *j* is not allowed), which are used to specifically analyse the change in the cumulative cases and healthcare resource congestion in the scenarios with different population migration rates. The results are shown in Figure 4 and Table 4 for the scenarios with different migration rates, and the detailed description of the results is described below.

**Table 4:** Sensitivity analysis results table.

**Group**	**Situation**	**Object**	**The final cumulative cases number**	**The daily growth rate of cumulative cases**	**The onset of healthcare resource congestion**	**The end of healthcare resource congestion**	**The duration of the congestion**
Taiyuan–Jinzhong	Migration occurs only from Jinzhong to Taiyuan (as shown by the green solid line in Figures 4e and 4f)	Taiyuan	Increases from 801,891 to 1,179,384 (1.47 times)	Rise (as shown by the slope of the green and purple solid lines in Figure 4e)	Does not change	Delayed (from 7th January 2023 to 11th January 2023)	Later by 4 days
	Migration occurs only from Taiyuan to Jinzhong (as shown by the blue solid line in Figures 4e, f)	Jinzhong	Decreases from 449,387 to 148,588 (0.33 times)	Decrease (as shown by the slope of green and purple solid lines in Figure 4f)	Does not change	Advanced (from 5th January 2023 to 26th December 2022)	Earlier by 10 days
		Taiyuan	Decreases from 801,891 to 655,893 (0.82 times)	Decrease (as shown by the slope of blue solid line in Figure 4e)	Does not change	Advanced (from 7 January 2023 to 5 January 2023)	Earlier by 2 days
	Population migration rate is reduced to 4/5 of the original value (as shown by the orange solid line in Figures 4e, f)	Jinzhong	Increases from 449,387 to 974,345 (2.17 times)	Rise (as shown by the slope of blue solid line in Figure 4f)	Advanced (from 17 December 2022 to 16 December 2022)	Delayed (from 5 January 2023 to 20 January 2023)	Later by 16 days
		Taiyuan	Increases from 801,891 to 832,926 (1.04 times)	Rise (as shown by the slope of orange solid line in Figure 4e)	Does not change	Does not change	Does not change
	No migration occurs between Taiyuan and Jinzhong (as shown by the red solid line in Figures 4e, f)	Jinzhong		Decrease (as shown by the slope of orange solid line in Figure 4f)	Does not change	Advanced (from 5 January 2023 to 3 January 2023)	Earlier by 2 days
		Taiyuan	Increases from 801,891 to 964,336 (1.20 times)	Rise (as shown by the slope of red solid line in Figure 4e)	Does not change	delayed (from 7 January 2023 to 9 January 2023)	Later by 2 days
		Jinzhong		Decrease (as shown by the slope of red solid line in Figure 4f)	Does not change	Advanced (from 5 January 2023 to 28 December 2023)	Earlier by 8 days
Linfen-Yuncheng	Migration occurs only from Yuncheng to Linfen (as shown by the green solid line in Figures 4g, h)	Linfen	Increases from 433,293 to 568,925 (1.31 times)	Rise (as shown by the slope of green solid line in Figure 4g)	Does not change	Delayed (from 3 January 2023 to 6 January 2023)	Later by 3 days
	Migration occurs only from Linfen to Yuncheng (as shown by the blue solid line in Figures 4g, h)	Yuncheng		Decrease (as shown by the slope of green solid line in Figure 4h)	Does not change	Advanced (from 4 January 2023 to 1 January 2023)	Earlier by 3 days
		Linfen	Decreases from 433,293 to 316,812 (0.73 times)	Decrease (as shown by the slope of blue solid line in Figure 4g)	Does not change	Advanced (from 3 January 2023 to 1 January 2023)	Earlier by 2 days
	Population migration rate is reduced to 4/5 of the original value (as shown by the orange solid line in Figures 4g, h)	Yuncheng		Rise (as shown by the slope of blue solid line in Figure 4h)	Does not change	Delayed (from 4 January 2023 to 12 January 2023)	Later by 8 days
		Linfen	Decreases from 433,293 to 431,408 (0.99 times)	Decrease (as shown by the slope of orange solid line in Figure 4g)	Does not change	Does not change	Does not change
	No migration occurs between Linfen and Yuncheng (as shown by the red solid line in Figures 4g, h)	Yuncheng		Rise (as shown by the slope of orange solid line in Figure 4h)	Does not change	Delayed (from 4 January 2023 to 5 January 2023)	Later by 1 days
		Linfen	Decreases from 433,293 to 398,924 (0.92 times)	Decrease (as shown by the slope of red solid line in Figure 4g)	Does not change	Advanced (from 3 January 2023 to 2 January 2023)	Earlier by 1 days
		Yuncheng		Rise (as shown by the slope of red solid line in Figure 4h)	Does not change	Delayed (from 4 January 2023 to 6 January 2023)	Later by 2 days

#### 3.4.1 Impact of population migration rate on cumulative cases

For Taiyuan city-Jinzhong city, (*I*) when the population migrates only from Jinzhong city to Taiyuan city (as shown by the green solid lines in Figures 4e, f), the final size of the cumulative number of infected people increases from 801,891 to 1,179,384 (1.47 times), and the rate of growth of the cumulative number of infected people per day increases in Taiyuan city as shown by the slopes of the green solid lines and the purple solid lines in Figure 4e; the cumulative number of infected people per day in Jinzhong city final size decreased from 449,387 to 148,588 (0.33 times), and the growth rate of the cumulative number of infected persons per day decreased as shown by the slopes of the green solid line and the purple solid line in Figure 4f. When the population migrates only from Taiyuan City to Jinzhong City (as shown by the blue solid lines in Figures 4e, f), the final size of the cumulative number of infected persons in Taiyuan City decreases from 801,891 to 655,893 (0.82 times), and the growth rate of the cumulative number of infected persons per day decreases as shown by the slopes of the blue and purple solid lines in Figure 4e; the final size of the cumulative number of infected persons in Jinzhong City increases from 449,387 to 974,345 (2.33 times), and the growth rate of the cumulative number of infected persons per day decreases as indicated by the slope of the green solid line and the purple solid line in Figure 4f. The final size of the cumulative number of infected persons in Jinzhong City increased from 449,387 to 974,345 (2.17 times), and the growth rate of the cumulative number of infected persons per day increased as shown by the slopes of the blue and purple solid lines in Figure 4f. (II) When the population migration rate is reduced to 4/5 of the original (as shown by the orange solid lines in Figures 4e, f), the final size of the cumulative number of infected people in Taiyuan City increases from 801,891 to 832,926 (1.04 times), and the growth rate of the cumulative number of infected people per day increases by the orange and purple solid lines in Figure 4e; the final size of the cumulative number of infected people in Jinzhong City decreases from 449,387 to 406,082 (0.90 times), and the growth rate of the daily cumulative number of infected persons decreases by the slopes of the orange and purple solid lines in Figure 4f. When there is no population mobility between Taiyuan City and Jinzhong City (as shown by the red solid lines in Figures 4e, f), the final size of the cumulative number of infected persons in Taiyuan City increases from 801,891 to 964,336 (1.20 times), and the growth rate of the cumulative number of infected persons per day increases from the slopes of the red and purple solid lines in Figure 4e; the final size of the cumulative number of infected persons in Jinzhong City decreases from 449,387 to decreased to 238,161 (0.53 times), and the growth rate of the cumulative number of infected persons per day decreased as shown by the slopes of the red and purple solid lines in Figure 4f.

For Linfen city-Yuncheng city, (I) when the population migration is only from Yuncheng city to Linfen city (as shown by the green solid line in Figures 4g, h), the final size of the cumulative number of infected people in Linfen city increases from 433,293 to 568,925 (1.31 times), and the growth rate of the cumulative number of infected people per day increases from the original as shown by the slopes of the green and purple solid lines in Figure 4g; and the cumulative number of infected people in Yuncheng city final size decreased from 710,766 to 530,526 (0.75 times), and the growth rate of the daily cumulative number of infected people decreased from the original one as shown by the slopes of the green and purple solid lines in Figure 4h. When the population migrated only from Linfen city to Yuncheng city (as shown by the blue solid line in Figures 4g, h), the final size of the cumulative number of infected people in Linfen city decreased from 433,293 to 316,812 (0.73 times), and the growth rate of the cumulative number of infected people per day was less than the original one as can be seen from the slopes of the blue and purple solid lines in Figure 4g; the final size of the cumulative number of infected people in Yuncheng city increased from 710,766 The final size of the cumulative number of infected people in Yuncheng City increased from 710,766 to 1,116,556 (1.57 times), and the growth rate of the cumulative number of infected people per day increased from the original by the slopes of the blue and purple solid lines in Figure 4h. (II) When the population migration rate was reduced to 4/5 of the original one (as shown by the orange solid lines in Figures 4g, h), the final size of the cumulative number of infected people in Linfen City decreased from 433,293 to 431,408 (0.99 times), and the growth rate of the cumulative number of infected people per day was reduced from the original one as shown by the slopes of the orange and violet solid lines in Figure 4g; in Yuncheng City, the final size of the cumulative number of infected people increased from 710,766 to 723,084 (1.02 times), and the growth rate of the cumulative number of infected people per day increased from the slope of the orange and violet solid lines in Figure 4h. When there is no population mobility between the two cities (as shown by the red solid line in Figures 4g, h), the final size of the cumulative number of infected people in Linfen City decreases from 433,293 to 398,924 (0.92 times), and the growth rate of the cumulative number of infected people per day decreases compared to the original one as shown by the slopes of the red and violet solid lines in Figure 4g; the final size of the cumulative number of infected people in Yuncheng City increases from 710,766 to 782,942 (1.10 times), and the growth rate of the daily cumulative number of infected persons increased from the original as shown by the slopes of the red and purple solid lines in Figure 4h.

#### 3.4.2 Analysis of the impact of migration rates on healthcare resource congestion

For Taiyuan–Jinzhong, (I) if the population only migrates from Jinzhong to Taiyuan (the results are shown as green solid lines in Figures 4e, f), the onset of healthcare resource congestion in Taiyuan and Jinzhong does not change, the end of healthcare resource congestion in Taiyuan is delayed (from 7th January 2023 to 11th January 2023) and the duration of the congestion is increased by 4 days compared to the original one; while in Jinzhong City the healthcare resource congestion ends earlier (from 5th January 2023 to 26th December 2022) and the duration of the congestion is 10 days less than it would otherwise be. Conversely, if the population only migrates from Taiyuan City to Jinzhong City (the results are shown as blue solid lines in Figures 4e, f), the onset of healthcare resource congestion in Taiyuan City remains unchanged, and the end of the congestion is brought forward (from 7 January 2023 to 5 January 2023), and the duration of the congestion is reduced by 2 days compared with the original one, whereas the onset of the healthcare resource congestion in Jinzhong City was brought forward (from 17 December 2022 to 16 December 2022), the end was delayed (from 5 January 2023 to 20 January 2023) and the duration of the congestion was increased by 16 days compared with the original one. (II) When the population migration rate in both places is reduced to 4/5 of the original level at the same time (the results are shown as orange solid lines in Figures 4e, f), the healthcare resource congestion in Taiyuan City will remain unchanged, but the onset time of healthcare resource congestion in Jinzhong City will remain unchanged, the end time of congestion will be earlier (from 5 January 2023 to 3 January 2023), and the duration of congestion will be reduced by 2 days compared with the original situation. In the case without population migration (results shown as red solid lines in Figures 4e, f), the onset time of healthcare resource congestion in Taiyuan and Jinzhong City remains unchanged compared to the actual situation, the end time of healthcare resource congestion in Taiyuan City is delayed (from 7 January 2023 to 9 January 2023), and the duration of congestion is increased by 2 days compared to the original situation; the end time of healthcare resource congestion in Jinzhong City is advanced (from 5 January 2023 to 28 December 2023) and the duration of congestion is reduced by 8 days.

For Linfen-Yuncheng, (I) only when population migration occurs from Yuncheng City to Linfen City (the results are shown as green solid lines in Figures 4g, h), the onset time of healthcare resource congestion remains unchanged in both cities, and the end of healthcare resource congestion is delayed in Linfen City (from 3 January 2023 to 6 January 2023), and the duration of healthcare resource congestion is increased by 3 days compared to the original one, while in Yuncheng City, the end of congestion is earlier (from 4 January 2023 to 1 January 2023) and the duration of healthcare resource congestion is 3 days shorter than the original one. If the population only migrates from Linfen City to Yuncheng City (the results are shown as blue solid lines in Figures 4g, h), the onset time of healthcare resource congestion in both cities remains unchanged, the end of healthcare resource congestion in Linfen City is advanced (from 3 January 2023 to 1 January 2023), and the duration of healthcare resource congestion is shortened by 2 days compared with the original one; However, the end of healthcare resource congestion in Yuncheng City is delayed (from 4 January 2023 to 1 January 2023), and the end of healthcare resource congestion in Yuncheng City is delayed (from 4 January 2023 to 12 January 2023), the duration of healthcare resource congestion is 8 days longer than the original one; (II) when the population migration rate in both places is simultaneously reduced to 4/5 of the original level (the results are shown as orange solid lines in Figures 4g, h), the healthcare resource congestion in Linfen City remains unchanged, while the onset time of healthcare resource congestion in Yuncheng City remains unchanged, the end time of healthcare resource congestion is delayed (from 4 January 2023 to 5 January 2023), and the duration of healthcare resource congestion is increased by 1 day compared with the original. In the case without population migration (results shown as red solid lines in Figures 4g, h), the onset time of healthcare resource congestion in the two cities remains unchanged, the end time of healthcare resource congestion in Linfen City is advanced (from 3 January 2023 to 2 January 2023), and the duration of healthcare resource congestion is reduced by 1 day compared with the original; in Yuncheng City, the end time of healthcare resource congestion is delayed (from 4 January 2023 to 6 January 2023) and the duration of healthcare resource congestion is increased by 2 days compared to the original.

## 4 Conclusion and discussion

During large-scale outbreaks of acute SARS-CoV-2, population mobility between different regions can lead to an increase in the number of cases, thus triggering a severe congestion of healthcare resources. This phenomenon has received extensive attention and in-depth study by many scholars (7–13). In this paper, the impact of inter-regional population mobility with different healthcare resource reserves and infections on the trend of epidemic and healthcare resource allocation were investigated using the example of SARS-CoV-2. The findings showed that the size of the cumulative cases in each city was ranked as Taiyuan > Jinzhong, Yuncheng > Linfen (Figures 4a–d purple solid lines), while the duration of healthcare resource congestion was ranked as Jinzhong > Taiyuan, Linfen > Yuncheng (Figures 4e, f purple solid lines). This suggests that although the demand for medical care is higher in areas with higher numbers of cases, it may not necessarily lead to more severe healthcare resource congestion. The severity of healthcare congestion not only depends on the number of infections and hospitalization demand, but is also influenced by factors such as local healthcare resource reserves. Since the healthcare resource reserve in Taiyuan (33,969 beds) was much higher than that in Jinzhong (21,216 beds), the duration of healthcare congestion was shorter than that in Jinzhong despite the fact that the number of cases in Taiyuan was higher than that in Jinzhong, and the same pattern applied to Linfen-Yuncheng. This finding is consistent with the findings of existing studies (26, 27). Sun et al. (26) used the XGBoost model to quantify the impact of various factors on the burden and clinical severity of SARS-CoV-2 and found that Shanghai's greater healthcare resources were likely an important reason for its lower SARS-CoV-2 infection rate. Barasa et al. (27) assessed the surge capacity of the Kenyan health system and found that regions with abundant healthcare resources showed greater resilience in responding to the SARS-CoV-2 epidemic.

In terms of the impact of population migration rate on the cumulative number of cases, unidirectional population migration control can reduce the cumulative number of cases in the migration area, while also leading to an increase in the cumulative number of cases in the migration area; second, bidirectional population migration control measures will increase the cumulative number of cases in cities with a higher number of cases, while leading to a decrease in the cumulative number of cases in cities with a lower number of cases; finally, when population mobility between regions stops completely, cities with a higher number of cases will experience a greater increase in the cumulative number of cases, while cities with a lower number of cases will experience a decrease in the cumulative cases.

Regarding the impact of migration rates on healthcare resource congestion, changes in migration rates have a small effect on the onset times of congestion in both pairs of cities (Taiyuan–Jinzhong and Linfen–Yuncheng), but they do influence the duration and end times of congestion. First, unidirectional population control can shorten the duration of healthcare resource congestion in the area from which the population is migrating and advance the end time of the congestion. However, this measure results in a prolonged duration of congestion and a delayed end time in the destination area. Second, bidirectional control of population migration affects the end times and duration of healthcare resource congestion. In cities with high numbers of cases, the end time of congestion is advanced and its duration is shortened, while the impact on cities with fewer cases is less pronounced. Finally, when migration between the two regions is completely stopped, cities with higher numbers of cases experience an increase in the duration of healthcare resource congestion and a delayed end time, while cities with fewer infections experience a reduction in congestion duration and an earlier end time. Our conclusions are in line with existing studies (28, 29). For example, Lai et al. (28), in a study evaluating the impact of travel restrictions in controlling interregional SARS-CoV-2 transmission, found that the cumulative number of cases could have increased 97-fold by 31 May 2020 if no interventions were implemented. Xue et al. (29) found that the cumulative number of cases would have increased by 290.1% between 24 February and 15 March 2020 if mainland China had not implemented travel bans. These results underscore the importance of travel control in limiting the spread of SARS-CoV-2. In summary, simply restricting the movement of people may not be effective in controlling the epidemic. It is essential to consider local healthcare resource reserves and infection situations to develop differentiated population control measures that can minimize the scale of infection and alleviate healthcare resource congestion.

This study investigates the dynamics of healthcare resource congestion under different population mobility scenarios in two groups of cities, using a healthcare resource dynamics model. The aim is to provide both theoretical insights and empirical evidence to support the optimization of population mobility management and epidemic prevention strategies. Simulation results reveal that while unidirectional population migration control or bidirectional population migration control measures can alleviate medical pressure in high-risk areas under certain conditions, they may also inadvertently increase strain on healthcare systems in low-risk regions—a phenomenon referred to as “second-generation congestion.” This finding highlights the limitations of traditional “one-size-fits-all” mobility control strategies, which may be inadequate in the face of multi-center, multi-wave outbreaks. To address this challenge, epidemic control strategies can be optimized in the following three aspects: firstly, the proposed inter-regional healthcare resource dynamics model—which integrates infection, migration, and congestion mechanisms—can be embedded into policy-making processes. By simulating the effects of population movement on healthcare supply and demand, the model helps to identify potential risk zones in advance and provides a scientific foundation for the proactive allocation of medical resources. Secondly, at the provincial or national level, a unified healthcare resource dispatching platform should be established to enable dynamic, cross-regional resource sharing. This would help form a “regional healthcare coordination circle,” enhancing the overall system's capacity to respond and reducing the risk of localized outbreaks. Finally, population mobility restrictions should be dynamically adjusted based on infection risk, healthcare system capacity, and the current stage of the epidemic, and this approach ensures that control measures are both precise and adaptive, balancing epidemic containment with the social impacts of mobility restrictions.

Compared to Feng et al.'s (30)research, we introduced Q, H and D groups (confirmed, hospitalized and deceased) to more accurately simulate the epidemic spread trend and make it more realistic. Compared to Fang et al.'s study (31), this research not only analyzed the dynamic changes of epidemic transmission under different population mobility scenarios in the target cities, but also examined their impact on healthcare resource congestion. This study has several limitations. Firstly, vaccination plays a crucial role in enhancing herd immunity. Future studies will explore in greater depth the impact of vaccination coverage and effectiveness on the overall herd immunity effect. Secondly, due to limitations in data availability, the empirical analysis in this study is currently restricted to urban areas in China. Future research could expand the dataset to include a broader range of countries and regions, particularly rural and remote areas, to further validate the robustness and generalizability of the model. Thirdly, several monitoring approaches, such as the statutory infectious disease reporting system and epidemiological follow-up conducted by disease control centers, can effectively track the movement of individual infected persons. These methods have been extensively utilized in the prevention and control of infectious diseases, thereby contributing to the refinement of epidemic prevention strategies and the optimization of medical resource allocation. In future research, we aim to further enhance data acquisition channels to improve the performance and accuracy of our model.

## Data Availability

The raw data supporting the conclusions of this article will be made available by the authors, without undue reservation.
